# Institutional courage buffers against institutional betrayal, protects employee health, and fosters organizational commitment following workplace sexual harassment

**DOI:** 10.1371/journal.pone.0278830

**Published:** 2023-01-25

**Authors:** Alec M. Smidt, Alexis A. Adams-Clark, Jennifer J. Freyd

**Affiliations:** 1 Center for Institutional Courage, Inc., Palo Alto, California, United States of America; 2 Department of Psychology, University of Oregon, Eugene, Oregon, United States of America; 3 Department of Psychiatry and Behavioral Sciences, Stanford University School of Medicine, Stanford, California, United States of America; University of East Anglia, UNITED KINGDOM

## Abstract

Workplace sexual harassment is associated with negative psychological and physical outcomes. Recent research suggests that harmful institutional responses to reports of wrongdoing–called *institutional betrayal*—are associated with additional psychological and physical harm. It has been theorized that supportive responses and an institutional climate characterized by transparency and proactiveness—called *institutional courage*—may buffer against these negative effects. The current study examined the association of institutional betrayal and institutional courage with workplace outcomes and psychological and physical health among employees reporting exposure to workplace sexual harassment. Adults who were employed full-time for at least six months were recruited through Amazon’s Mechanical Turk platform and completed an online survey (*N* = 805). Of the full sample, 317 participants reported experiences with workplace sexual harassment, and only this subset of participants were included in analyses. We used existing survey instruments and developed the Institutional Courage Questionnaire-Specific to assess individual experiences of institutional courage within the context of workplace sexual harassment. Of participants who experienced workplace sexual harassment, nearly 55% also experienced institutional betrayal, and 76% experienced institutional courage. Results of correlational analyses indicated that institutional betrayal was associated with decreased job satisfaction, organizational commitment, and increased somatic symptoms. Institutional courage was associated with the reverse. Furthermore, results of multiple regression analyses indicated that institutional courage appeared to attenuate negative outcomes. Overall, our results suggest that institutional courage is important in the context of workplace sexual harassment. These results are in line with previous research on institutional betrayal, may inform policies and procedures related to workplace sexual harassment, and provide a starting point for research on institutional courage.

## Introduction

The recent emergence of the #MeToo movement has underscored the pervasiveness of sexual harassment and related workplace misconduct. Although public awareness of these issues has recently exploded, brave individuals (e.g. Anita Hill) have many times raised the issue, and researchers have for decades investigated workplace sexual harassment, with some characterizing it as, “still the last great open secret” [1]. Decades of empirical research substantiate the high rates of sexual harassment exposed by public #MeToo discourse; multiple studies have found that a significant percentage of employees experience sexual harassment in the workplace, with women bearing the brunt of victimization (estimates between nearly 50% to 75% for women and nearly 15% to 30% for men [2, 3]).

Sexual harassment is not without significant costs. In prior research, workplace sexual harassment has been found to affect employee and workplace outcomes at multiple levels. At the micro level, workplace sexual harassment is associated with lower overall job satisfaction, lower satisfaction with co-workers and supervisors, lower life satisfaction, and decreased psychological and physical well-being among individual employees [4]. At the macro, institutional level, workplace sexual harassment is associated with decreased employee organizational commitment and greater withdrawal from workplace duties, among others [4, 5]. The harms of sexual harassment are particularly notable, given research suggesting that sexual harassment may be more harmful than other types of workplace aggression [6].

In addition to the harm caused by direct exposure workplace sexual harassment, simply existing within a hostile workplace institution where such behavior occurs may be associated with unique and/or additional harm. For instance, Hulin, Fitzgerald, & Drasgow [7] found that even indirect exposure to sexual harassment (e.g., witnessing coworkers be harassed) and a climate of organizational tolerance for sexual harassment (e.g., an environment that does not take seriously reports of harassment) were related to negative psychological and workplace outcomes, even after accounting for direct exposure to direct sexual harassment [8, 9]. In a cyclical fashion, such a climate is also related to a higher sexual harassment incidence rate.

## Institutional betrayal

Although the existing research regarding the organizational context of sexual harassment is important, additional research is needed to examine how institutional actions and/or inactions may exacerbate the harm of an individuals’ experiences of workplace sexual harassment, particularly given many employees’ dependence on their employer. *Institutional betrayal* [10] is a useful framework for conceptualizing the harms of workplace sexual harassment. The concept of institutional betrayal arose out of over 20 years of research on betrayal trauma theory [11]. Betrayal trauma theory posits that trauma perpetrated by an individual’s close and trusted other will be less available to awareness and memory and will result in greater negative outcomes than trauma perpetrated by someone unknown to the individual [11]. Close and trusted others can include caregivers, family members, intimate partners, and other individuals on whom the victim depends for resources, support, and, in some cases, survival. A number of studies on betrayal trauma theory have found that, indeed, trauma perpetrated by a close and trusted other is associated with greater unawareness, as well as negative psychological and physical health outcomes, compared to trauma perpetrated by someone unknown to the victim [12–15].

As with interpersonal betrayal trauma, so too is there a relationship between individuals and the institutions on which they depend for resources, support, protection, and at times survival. Institutional betrayal occurs when such institutions (e.g., universities, the military, organized religion, and public/private corporations) intentionally or negligently harm their members, breaching this relationship of trust and dependence. The term institutional betrayal was first offered by Freyd [16] and further explicated by Platt, Barton, and Freyd [17] to describe such violations. The first empirical investigation of institutional betrayal was by Smith and Freyd [18], who examined responses to sexual assault on college campuses. Smith and Freyd [18] found that approximately 45% of women who experienced sexual assault also experienced institutional betrayal. Experiencing institutional betrayal was associated with greater anxiety, dissociation, and sexual problems compared to individuals who had not experienced institutional betrayal. Numerous other studies have since replicated the link between institutional betrayal and exacerbated mental and physical health symptoms in samples of university students [19–22], as well as in healthcare [23, 24] and military [25] settings. In addition, Brewer and colleagues investigated institutional betrayal in the context of workplace bullying among nurses.

Research thus far on institutional betrayal has not adequately examined workplace sexual harassment, even though employees who experience sexual harassment may be particularly at risk for institutional betrayal. Employees who experience sexual harassment within the context of their employment organization exist in the difficult bind frequently described by betrayal trauma theory and institutional betrayal. Employees not only depend upon and trust their employer to protect and adjudicate complaints of sexual harassment effectively, but also depend upon them for a variety of other resources, including their income and health insurance. Additional harm may be caused when an employer breaches the trust of its employee by ineffectively preventing or responding to sexual harassment within its organization. In other words, the harm of sexual harassment may not only be related to what happened, but also about what happened next. No prior research to our knowledge has investigated how institutional betrayal may exacerbate the psychological, physical, or workplace outcomes of sexual harassment.

## A need for institutional courage

Clearly, experiences of institutional betrayal are associated with harm and are not rare occurrences. Previous research on institutional betrayal suggests that institutions often respond negatively to reports of harm or misconduct, and these types of responses are harmful to members of their institutions. It is the next logical step, then, to investigate how and if there are institutional behaviors that may replace institutional betrayal and/or counter the effects of institutional betrayal. We call these types of institutional behaviors *institutional courage* [26], and they are the “antidote” to institutional betrayal. Institutional courage is “accountability, transparency, actively seeking justice, and making reparations where needed” [27].

Freyd [28] first articulated the principles of institutional courage, which include institutional behaviors such as supportively responding to victims and whistleblowers, engaging in self-study, and a culture of transparency at all levels. Smidt and Freyd [27] noted that as betrayal occurs at multiple levels, so too can courage occur at both the interpersonal and institutional level. As prior research on institutional betrayal clearly demonstrates the impact that an institutional response has on individuals who report harm [18–21], a first step is to investigate institutional courage following individual incidents misconduct, such as sexual harassment, that occurs in an institutional setting.

## Current study

The current study expands on prior institutional betrayal research and examines a novel, but related concept—institutional courage. First, we examine an additional context where institutional betrayal is likely present—workplace sexual harassment. Second, we measure and systematically examine the concept of institutional courage. The overall goal of the current study is to determine how institutional courage and institutional betrayal following experiences of workplace sexual harassment are associated with employee workplace and employment-related outcomes, psychological health outcomes, and physical health outcomes. The central hypothesis of this study was that, among participants reporting experiences with workplace sexual harassment, institutional courage will be associated with more positive employee workplace and employment-related outcomes (H1), psychological health outcomes (H2), and physical health (H3), whereas institutional betrayal will be associated with more negative employee workplace and psychological/physical health outcomes. We predicted that these variables would be associated, even when controlling for gender and prior trauma history unrelated to sexual harassment. We also predicted that institutional courage and institutional betrayal will interact, such that institutional courage will attenuate the relationship between institutional betrayal and both employee workplace (H4) and psychological/physical health outcomes (H5). Theoretically and empirically, institutional *betrayal* consists of harmful actions that have real impacts, psychologically and physically. And conversely, institutional *courage* consists of supportive actions that would have the opposite effect. Thus, we predict an attenuation effect when both are present. In this study, we examined the following employee workplace and employment-related outcomes: job satisfaction, trust in management, organizational commitment, work withdrawal behaviors, one-year intent to remain at the institution, and perceived institutional gender equality. We also examine psychological health outcomes (depression, anxiety) and a physical health outcome (somatic symptoms).

## Method

### Participants

Study participants were recruited from Amazon Mechanical Turk (MTurk). MTurk is an online labor market that allows individuals to complete brief tasks, or HITs (Human Intelligence Tasks), for pay. MTurk workers are aged at least 18 years and are based in a variety of countries. US MTurk workers are largely representative of the United States general population, but tend to skew younger, be slightly more educated, and are more likely to identify as European-American and Asian-American [29–31]. Approximately 57% MTurk workers report currently being employed, working nearly 37 hours on average per week [31]. Workers also are employed in a variety of industries that are distributed relatively closely to large-scale national, stratified surveys (e.g., the Congressional Cooperative Election Survey; [30]). To ensure data quality, we only recruited MTurk workers who had a HIT acceptance rate (HAR) of greater than or equal to 95%, along with at least 50 approved HITs. These steps help ensure data quality and have been used previously in a number of studies using the MTurk platform (e.g., [31, 32]).

To ensure data integrity, participants were also required to answer no more than one “attention check” question incorrectly (e.g., “I will choose ‘Agree’ if I am paying attention”) or their participation would be discontinued. During initial data collection, participants completed four attention check questions. Additional, more sophisticated attention check questions were systematically added throughout data collection when concerns arose about possible participation of non-human actors that attempt to mimic human responses to survey measures, commonly referred to as “bots.” The majority of the sample was collected using six attention check questions (74.4%; *n* = 599). The remainder of the sample answered either answered five (13.2%; *n* = 106) or four (12.4%; *n* = 100) attention check questions.

A total of 971 participants were recruited and completed the study in full. Inclusion criteria for participation in the current study were an age of at least 18, current full-time employment for at least six months, and residence in the United States. Participants who did not meet these criteria were excluded from the study (*n* = 10), as well as participants who provided illogical responses (e.g., reporting an age of 30 and also reporting military service during the Vietnam War; *n* = 54), nonsensical responses (e.g., unrelated content in open-ended responses; *n* = 11), or “straight-lined” responses (*n* = 11). Participants were also excluded for failing a manipulation check question regarding an experiment that was included in the study (not reported on in this paper [33]; *n* = 36), or for failing to report demographic or employment information (*n* = 44). Of the 971 participants who completed the study, 805 participants provided data that met our data inclusion criteria and were used in data analysis.

Participants in the final sample were an average age of 32.35 years (*SD* = 10.18), and women and men were approximately equally represented. The majority of participants identified as heterosexual/straight (87%) and White (75.5%). A plurality of participants had a bachelor’s degree (41.2%), with 90.4% of the sample reporting an income range above the federal poverty line. The modal income level for this sample was at the $50,000 and $59,000 range. and the average yearly salary was $47, 908 (*SD* = $28,052). Participants worked in a range of industries and departments, and the majority were employed in the private for-profit sector (75.8%). The majority of participants identified as non-management, individual contributor employees (57.1%). On average, participants had been at their current place of employment for approximately six and a half years (*M* = 6.51, *SD* = 5.48) and worked on average just over 40 hours per week (*M* = 41.85, *SD* = 5.35). See Table 1 for full demographic information and Table 2 for full employment information for both the full sample (*N* = 305) and the subsample used analyses (*n* = 317). The demographic characteristics of the subsample reporting sexual harassment was relatively comparable compared to the full sample.

**Table 1:** Demographic information of full sample (*N* = 805) and subset of sample (*n* = 317) used in analyses.

Gender Identity	*n*/805	%	*n*/317	%	Education	*n*/805	%	*n*/317	%
Woman	378	47.0	168	53.0	Bachelor’s degree	332	41.2	138	43.2
Man	411	51.1	140	44.2	Some college	159	19.8	67	21.1
Transwoman	5	0.6	4	1.3	Master’s degree	97	12.0	42	13.2
Transman	2	0.2	1	0.3	High school diploma/GED	85	10.6	29	9.1
Genderqueer	4	0.5	3	0.9	Associate degree	81	10.1	25	7.9
Non-binary gender	4	0.5	1	0.3	Vocational/technical school	26	3.2	9	2.8
Another gender	1	0.1	0	0.0	Doctoral degree	11	1.4	3	0.9
**Gender Identity at Birth**					Professional degree	9	1.1	2	0.6
Same GID now as at birth	790	98.1	307	96.8	Less than high school	5	0.6	2	0.6
Different GID now as at birth	15	1.9	10	3.2	**Income**				
**Sexual Orientation**					$50,000 to $59,999	113	14.0	43	13.6
Heterosexual	703	87.3	264	83.3	$30,000 to $39,999	112	13.9	48	15.1
Bisexual	39	4.8	21	6.6	$60,000 to $69,999	86	10.7	32	10.1
Lesbian	23	2.9	15	4.7	$40,000 to $49,999	85	10.6	33	10.4
Gay	16	2.0	6	1.9	$100,000 to $149,999	84	10.4	27	8.5
Asexual	7	0.9	2	0.6	$20,000 to $29,999	77	9.6	42	13.2
Another sexual orientation	7	0.9	2	0.6	$70,000 to $79,999	67	8.3	26	8.2
Queer	6	0.7	4	1.3	$10,000 to $19,999	53	6.6	27	8.5
Questioning	4	0.5	3	0.9	$90,000 to $99,999	44	5.5	10	3.2
**Race**					$80,000 to $89,999	39	4.8	13	4.1
Caucasian	608	75.5	231	72.9	$150,000 or more	37	4.6	11	3.5
Black/African American	63	7.8	35	11.0	Less than $10,000	8	1.0	5	1.6
Asian/Asian American	47	5.8	16	5.0	**Ethnicity**				
Latino American	44	5.5	12	3.8	Non-Hispanic/Spanish/Latino	736	91.4	291	91.8
Multiracial	37	4.6	20	6.3	Hispanic	45	5.6	16	5.0
Another racial identity	2	0.2	1	0.3	Latino	14	1.7	5	1.6
American Indian/Native American	2	0.2	1	0.3	Hispanic and Latino	5	0.6	3	0.9
Native Hawaiian/Pacific Islander	1	0.1	1	0.3	Spanish	3	0.4	1	0.3
Middle Eastern	1	0.1	0	0.0	Hispanic and Spanish	2	0.2	1	0.3

**Table 2:** Employment information for full sample (*N* = 805) and subset of sample (*n* = 317) used in analyses.

Number of employees	*n*/805	%	*n*/317	%	Department	*n*/805	%	*n*/317	%
100–249	114	14.2	47	14.8	Customer Service	152	18.9	62	19.6
50–99	107	13.3	34	10.7	Other	110	13.7	43	13.6
20–49	105	13.0	48	15.1	IT	105	13.0	40	12.6
250–499	101	12.5	47	14.8	Administrative	98	12.2	35	11.0
5000+	82	10.2	32	10.1	Sales	72	8.9	23	7.3
500–999	75	9.3	30	9.5	Product	35	4.3	16	5.0
1000–5000	71	8.8	20	6.3	Finance	33	4.1	16	5.0
10 to 19	61	7.6	24	7.6	Research & Development	33	4.1	11	3.5
5 to 9	47	5.8	19	6.0	Manufacturing	31	3.9	15	4.7
2 to 4	29	3.6	12	3.8	Marketing Operations	24	3.0	10	3.2
1	13	1.6	4	1.3	Engineering	24	3.0	7	2.2
**Employment industry**					Accounting	23	2.9	12	3.8
Profession, Scientific, and Technical Services	96	11.9	25	7.9	Human Resources	21	2.6	9	2.8
Retail Trade	89	11.1	32	10.1	Legal	16	2.0	3	0.9
Health Care and Social Assistance	78	9.7	36	11.4	Public Relations	14	1.7	9	2.8
Finance and Insurance	75	9.3	36	11.4	Business Intelligence	11	1.4	5	1.6
Education Services	69	8.6	26	8.2	International	3	0.4	1	0.3
Information	61	7.6	23	7.3	**Job level**				
Administrative and Support	48	6.0	14	4.4	Indiv contributor (non-manager)	460	57.1	175	55.3
Manufacturing	47	5.8	19	6.0	Manager (>3 years experience)	155	19.3	68	21.5
Arts, Entertainment, and Recreation	40	5.0	22	6.9	Manager (<3 years experience)	94	11.7	43	13.6
Hotel Accommodation and Food Services	38	4.7	20	6.3	Other	57	7.1	17	5.4
Other Services (except Public Admin)	34	4.2	11	3.5	Leader	24	3.0	7	2.2
Construction	26	3.2	9	2.8	Executive/C-Suite	15	1.9	7	2.2
Transportation and Warehousing	24	3.0	10	3.2					
Real Estate and Rental and Leasing	17	2.1	8	2.5					
Public Administration	16	2.0	6	1.9					
Wholesale Trade	15	1.9	5	1.6					
Utilities	10	1.2	7	2.2					
Management of Companies and Enterprises	9	1.1	2	0.6					
Military	7	0.9	4	1.3					
Agriculture, Forestry, Fishing and Hunting	5	0.6	2	0.6					

## Materials

### Workplace sexual harassment

Workplace sexual harassment was assessed using the Sexual Experiences Questionnaire-Department of Defense Workplace Gender Relation Survey (SEQ; [34]). The SEQ is a 23-item measure that assesses experiences of different types of sexual harassment at work during the past year using four subscales. Subscales include: sexist hostility (e.g., “treated you ‘differently’ because of your sex [for example, mistreated, slighted, or ignored you]”); sexual hostility (e.g., “repeatedly told sexual stories or jokes that were offensive to you”); unwanted sexual attention (e.g., “touched you in a way that made you feel uncomfortable”); and sexual coercion (e.g., “treated you badly for refusing to have sex”). In addition to the standard items, we included four additional items that are part of revised versions of the SEQ [34]. Participants responded to all items using a 5-point Likert scale (1 = “Never,” 5 = “Many times”). The SEQ was scored by creating a total summed score of all items and calculating incidence rates (i.e., the percentage of individuals experienced at least one instance of sexual harassment). The SEQ is one of the most widely used measures of sexual harassment [5] and demonstrates good internal consistency [34]. In the current study, this measure demonstrated good internal consistency (α = .97).

### Institutional betrayal

Institutional betrayal was assessed with the Institutional Betrayal Questionnaire-Version 2 (IBQ; [21]). The IBQ is a 12-item measure that assesses different experiences of institutional betrayal. Given that experiencing institutional betrayal requires first experiencing an index event (e.g., an employee is sexually harassed by her boss [the index event] and is subsequently punished in some way for reporting the experience [the subsequent institutional betrayal]), participants were presented with the IBQ following completion of the SEQ. Importantly, and given the necessity of experiencing an index event, only participants who endorsed at least one item on the SEQ were shown the IBQ. Example items include: “covering up the experience” and “punishing you in some way for reporting the experience (e.g., loss of privileges of status).” Response options included: “Yes,” “No,” and “Not applicable.” Scores on the IBQ were determined by summing the number of items endorsed with “Yes,” with a theoretical score range of 0–12. As with the SEQ, we calculated incidence rates (i.e., the percentage of individuals who have experienced at least one instance of institutional betrayal).

### Institutional courage

Institutional courage was assessed with the Institutional Courage Questionnaire–Specific (ICQ-Specific; [35]). The ICQ-Specific is an 18-item measure designed to assess experiences of institutional courage that was created for the purposes of this study. The ICQ-Specific was presented to participants along with the IBQ following their completion of the SEQ. This measure, like the IBQ, is designed to be completed only by those participants who have experienced workplace sexual harassment. Participants were given the same instructions and response options as the IBQ. Example items include: “creating an environment where continued employment was not more difficult for you than before the experience occurred” and “not covering up the experience.” In line with the IBQ scoring, scores on the ICQ-Specific were determined by summing the number of items endorsed with “Yes,” with a theoretical score range of 0–18. We will also calculate incidence rates for institutional courage (i.e., the percentage of individuals who have experienced at least one instance of institutional courage).

### Perceived gender bias

Perceptions of gender bias in participants’ current workplace were assessed using four items [36]. Participants rated their agreement on a 7-point Likert scale (1 = “Strongly disagree,” 7 = “Strongly agree”) with statements about whether men and women are treated equally in the workplace (e.g., “I think women and men are treated the same way at my current place of employment”). Items were averaged to create a composite score that represents participants’ perceptions of gender bias in their current workplace, with higher scores indicating less perceived gender bias. In both the Kaiser and colleagues [36] and another study [33], these four items demonstrated good internal consistency (α = .88 and α = .95, respectively). In the current study, these items demonstrated good internal consistency (α = .94).

### Job satisfaction

The Abridged Job in General Scale (aJIG; [37]) is an 8-item scale that measures global job satisfaction. Participants were instructed to consider their jobs in general and select “Yes,” “No,” or “?” for each item. Items are either single words or brief phrases that might describe participants’ current jobs, including: “Good,” “Undesirable,” and “Makes me content.” A composite score is created by summing each of the eight items. The aJIG is an abridged version of the full-length 18-item Job in General Scale developed by Ironson and colleagues [38]. The aJIG demonstrated good internal consistency across three studies during its initial development [37]. In the current study, this measure demonstrated good internal consistency (α = .91).

### Organizational commitment

Organizational commitment was assessed used the Organizational Commitment Questionnaire (OCQ; [39]). The OCQ was used to assess affective organizational commitment using the 6-item affective organizational commitment subscale. Affective organizational commitment refers to one’s affective or emotional attachment to and identification with an organization. An example item is “I really feel as if this organization’s problems are my own.”. The measure is scored by taking the average of the each of the six items, and has demonstrated good internal consistency across previous studies [40] and the current study (α = .94).

### One-year leaving intentions

Two items from the Staying or Leaving Index (SLI; [41]) were used to assess participants’ one-year intentions to leave their current place of employment. Participants responded to two questions: “How do you rate your chances of still working at your current place of employment?” and “How would you rate your chances of quitting your current place of employment?” Participants responded using a 7-point Likert scale (1 = “Definitely unlikely,” 7 = “Definitely likely”). The SLI demonstrated good internal consistency in past research [41] and the current study (α = .89).

### Work withdrawal behaviors

Work withdrawal behaviors were assessed using 19 items from Hanisch and Hulin’s (WJW; [42]) measure of work withdrawal. This measure assesses work withdrawal behaviors, broadly conceptualized as avoidance of or actual disengagement from day-to-day work activities (e.g., “Wandering around looking busy so you do not have to do your work?”). Participants used an 8-point Likert scale (1 = “Never in the past year,” 8 = “More than once in the past week”) to report how frequently they engage in these behaviors. The WJW has demonstrated good internal consistency in previous studies [42, 43]. In the current study, this measure demonstrated good internal consistency (α = .81).

### Trust in management

The Trust in Management Scale (TIM; [44]) is a 12-item measure used to assess employee trust in institutional management. Participants were instructed to consider the managers and executives at their workplace and select “Yes,” “No,” or “?” for each item. A composite score is created by summing each of the 12 items. Items are either single words or brief phrases that might describe management and executives in a given organization. Example word or phrases include: “Qualified,” “Concerned for employees’ welfare,” and “Consistent.” The TIM has demonstrated good internal consistency and validity [44]. In the current study, this measure demonstrated good internal consistency (α = .91).

### Depression, anxiety, and somatic symptoms

The Patient Health Questionnaire (PHQ; [45]) is a multi-subscale questionnaire that assesses multiple aspects of psychological and physical health. For this study, the depression subscale (PHQ-9), the anxiety subscale (GAD-7), and the somatic symptom subscale (12 items) were used to assess psychological health (depression symptoms and anxiety symptoms) and physical health (somatic symptoms). Participants report how often they experience each symptom using a 3-point Likert scale (0 = “Not bothered at all,” 3 = “Bothered a lot”) within a specified timeframe (within the last two weeks for depression and anxiety symptoms and within the last four weeks for somatic symptoms). A composite score was computed for each subscale by summing all items within each subscale. The PHQ and its subscale have demonstrated good internal consistency among a number of studies [45–47] and has been previously used in research on institutional betrayal [19]. In the current study, these subscales demonstrated good internal consistency (symptoms: α = .88; anxiety symptoms: α = .91; and somatic symptoms: α = .85; depression).

### Lifetime trauma history

The Brief Betrayal Trauma Survey-14 (BBTS; [48]) was used to assess lifetime trauma history. The BBTS is a 28-item measure that assesses traumatic experiences, ranging from those that are low in interpersonal betrayal (e.g., “been in a major earthquake, fire, flood, hurricane, or tornado that resulted in significant loss of personal property, serious injury to yourself or a significant other, the death of a significant other, or the fear of your own death.”) to those high in interpersonal betrayal (e.g., “you were made to have some form of sexual contact, such as touching or penetration, by someone with whom you were very close”). Participants indicate how many times (“Never,” “One or two times,” or “More than that”) they have experienced each of the 14 events both before and after the age of 18. Items were scored by binning each item into one of three categories: low, medium, or high betrayal; this yielded three variables that were then used in the analyses.

### Employment information

Participants were asked a series of questions about their current place of employment, including number of employees, employment sector (e.g., public vs. private, non-profit vs. for-profit), employment industry, current occupation (e.g., management vs. office and administrative support), department of their position (e.g., accounting vs. IT), job title (e.g., individual contributor/non-management vs. executive/C-suite), hours worked per week, number of years at current place of employment, number of years in current role, and current yearly salary. Questions about number of employees, employment sector, employment industry, and occupation were based on the Congressional Cooperative Election Survey [49], a large-scale national, stratified survey that has been used to compare MTurk participant demographics to those of participants in national stratified surveys [30]. Considering that it was possible that a portion of participants may work for multiple employers, participants were asked about the number of employers for whom they current work. If they indicated they worked for more than one employer, they were asked to choose one as their primary employer to report on.

### Demographic information

Participants answered a number of questions about their demographic information, including age, gender identity (both current and as assigned at birth), sexual orientation, race and ethnicity, highest level of schooling, current household income, and country of residence.

## Procedure

A HIT was posted to the Amazon MTurk site, and potential participants were asked to participate in a web-based study. Participants who signed up for the study were directed to click a link leading to brief screening questionnaire. Following completion of the screening questionnaire, eligible participants were directed to the main survey battery hosted by Qualtrics, which contained an online informed consent form. We used the method within Qualtrics described by Burleigh, Kennedy, and Clifford [50] to prevent participation by individuals using a Virtual Private Network (VPN), proxy server, or a non-United States IP address.

Participants clicked that they had read, understood, and agreed to the information on the informed consent form, including a warning that their system would be checked for the presence of a VPN/proxy/non-US IP address. Within five days of completing the study, participants were paid through the Amazon Payments system.

Initially, participants were compensated four dollars for their participation and completion of the study in its entirety. However, feedback from participants indicated that some participants believed that the compensation should be raised given the amount effort expended. To reduce burden on participants and be responsive to feedback, compensation was raised to five dollars, with the majority of participants in the final sample being compensated under the five-dollar compensation scheme (72.3%; *n* = 582). All study procedures were approved by the University of Oregon Office of Research Compliance (Institutional Review Board). Informed consent was obtained from all participants prior to study procedures.

## Results

### Missing data

Data were examined for missingness before proceeding with analyses. Missing data >5% was found on the following outcomes of interest: PHQ Depression Subscale (16.6%), BBTS (15.3%), and PHQ Anxiety Subscale (12.0%). Missing values were imputed using SPSS Version 25 Multiple Imputation using 10 imputed datasets. Measures of lifetime trauma history (BBTS), institutional betrayal (IBQ), and institutional courage (ICQ-Specific) were not imputed. These measures assess the presence or absence of certain experiences and concerns arose about both the lack of empirical support for imputation of these measures and the accuracy of imputation regarding these types of experiences. The majority of the missingness on the BBTS was related to an error in the Qualtrics survey software. At least one participant noted that there were problems with the display of this measure, as it appeared to be too wide on smaller screens. An examination of the missingness patterns of the BBTS revealed that the majority of missingness occurred in the “age 18 and older” items that were to the rightmost side of the screen that were occluded. Following imputation and computation of the below tests (i.e., linear regression, *t*-tests, etc.), the *micombine* function (version 1; part of the mice/miceadds package; [51] in *R* (version 3.5.3)) was used to pool the estimates of *F* tests using the *D*^*2*^ statistic, which provided the pooled *F*-statistic and *p*-value, as well as the *D*^*2*^-adjusted degrees of freedom [51, 52].

### Examination of the ICQ-Specific

We subjected the ICQ-Specific to a principal component analysis (PCA) to determine if the ICQ-Specific captured a unidimensional construct that could be defined as institutional courage. This was a similar approach taken by Smith and Freyd [18] using the IBQ, who found that the IBQ is best utilized as a one-dimensional structure. The results of the PCA suggest that the ICQ-Specific is best utilized as a one-dimensional structure. A one-component structure had an eigenvalue of 8.77 that explained 48.71% of the variance.

### Sexual harassment and gender differences

Across participants, 39.37% (*n* = 317) reported at least one instance of workplace sexual harassment. Forty-four percent of women (*n* = 168) reported at least one instance of sexual harassment, with men at 34.10% (*n* = 140), transwomen at 80% (*n* = 4), genderqueer participants at 75% (*n* = 3), transmen at 50% (*n* = 1), and non-binary participants at 25% (*n* = 1). Due to small cell sizes, participants with a gender identity other than “man” or “woman” (*n* = 9) were excluded from gender analyses. Women were more likely to experience at least one instance of sexual harassment than were men (χ^2^ [1, *N* = 789] = 8.92, *p* = .003). Of those participants who did experience sexual harassment, women had higher scores on the SEQ (*M* = 10.68, *SE* = 1.14) compared to men (*M* = 10.35, *SE* = 1.28), but these differences were not statistically significant, *t*(284.865) = 0.20, *p* = .844. The analyses presented in this paper use data from only these 317 participants who reported at least one instance of sexual harassment.

### Institutional betrayal prevalence

Six participants (1.9%) did not complete the IBQ following their completion of the SEQ. Of participants who experienced sexual harassment, 54.64% (*n* = 165) reported at least one experience of institutional betrayal. With respect to gender differences, 53.61% (*n* = 89) of women and 55.88% (*n* = 76) of men who reported sexual harassment also reported at least one experience of institutional betrayal; however, these differences were not statistically significant (χ^2^ [1, *N* = 302] = 0.11, *p* = .654). Of those participants that did experience institutional betrayal, women experienced significantly more types of institutional betrayal (*M* = 4.83, *SE* = 0.37) compared to men (*M* = 3.70, *SE* = 0.31; *t*[161.330] = 2.37, *p* = .018).

### Institutional courage prevalence

Ten participants (3.2%) did not complete the ICQ-Specific following their completion of the SEQ. Of participants who experienced sexual harassment, 76.17% reported at least one experience of institutional courage. With respect to gender differences, 71.34% of women (*n* = 117) and 82.09% of men (*n* = 110) who reported sexual harassment also reported at least one experience of institutional courage, and these differences were statistically significant (χ^2^ [1, *N* = 298] = 4.69, *p* = .030). Of those participants who did experience institutional courage, women experienced more types of institutional courage (*M* = 8.01, *SE* = 0.53) compared to men (*M* = 6.97, *SE* = 0.49), but these differences were not statistically significant, *t*(224.657) = 1.44, *p* = .151.

### Workplace outcomes—H1 & H4

#### Correlational results—H1

Bivariate Pearson’s *r* correlations were computed to examine the initial associations between institutional betrayal, institutional courage, and employee workplace outcomes among the subset of participants who reported sexual harassment (*n* = 317). Institutional betrayal was negatively correlated with job satisfaction, affective organizational commitment, and perceptions of gender bias (higher scores indicate lower perceptions of gender bias). Institutional betrayal was positively correlated with work withdrawal behaviors and one-year leaving intention (lower scores indicate lower intentions of leaving one’s current employer). Institutional betrayal was not correlated with trust in management (Table 3).

**Table 3:** Means, standard deviations, and bivariate correlations between IBQ, ICQ-Specific and employee workplace outcomes (*N* = 317).

Variable	*M*	*SD*	1	2	3	4	5	6	7
1. Institutional betrayal	2.36	3.15							
2. Institutional courage	5.78	5.70	-.08						
3. Job satisfaction	15.89	8.36	-.36***	.36***					
4. Affective commitment	4.12	1.54	-.34***	.34***	.72***				
5. Perceived gender bias	4.85	1.62	-.35***	.25***	.44***	.34***			
6. One-year leaving intentions	5.87	3.31	.31***	-.24***	-.57***	-.58***	-.21***		
7. Work withdrawal	49.07	18.42	.14*	-.14*	-.35***	-.34***	-.10	.32***	
8. Trust in management	17.52	3.61	.00	.14*	.12*	.15**	.11	-.09	-.01

*Note*. *M* and *SD* are used to represent mean and standard deviation, respectively.

*p < .05.

**p < .01.

***p < .001.

Institutional courage was negatively correlated with work withdrawal behaviors and one-year leaving intentions (lower scores indicate lower intentions of leaving one’s current employer). Institutional courage was positively correlated with job satisfaction, trust in management, affective organizational commitment, and perceptions of institutional gender bias (higher scores indicate lower perceptions of gender bias; see Table 3). Institutional betrayal and institutional courage were not significantly correlated with each other.

#### Regression results—H4

In order to examine our hypotheses regarding an attenuation effect between institutional courage and institutional betrayal on workplace outcomes among those experiencing sexual harassment, hierarchical linear multiple regression was used to examine the unique role of institutional betrayal, institutional courage, and their interaction on each of the employee workplace outcome variables of interest, controlling for gender. We included gender as a covariate (with cisgender women as the reference group) because of evidence suggesting that gender is linked to higher rates of sexual harassment and perceptions of gender bias. In addition, women reported significantly higher scores on the IBQ in this study. Each employee workplace variable was entered as the dependent variable. Gender and the mean-centered IBQ and ICQ variables were entered in the first step, followed by their interaction term in the second step. Participants who did not complete either the IBQ or ICQ were excluded from regression analyses.

**Fig 1:**
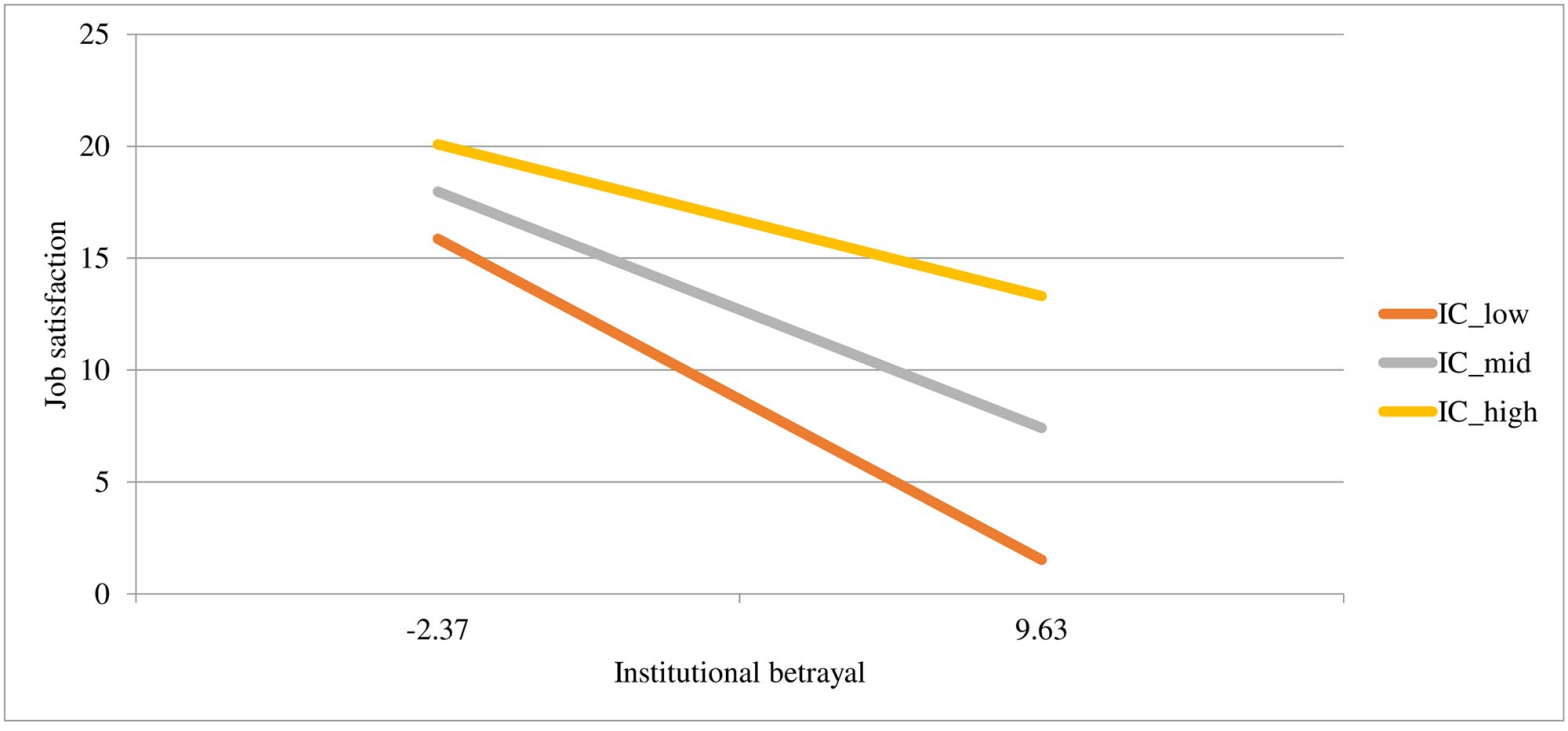
Associations between different levels of institutional courage (at -1SD, the mean, and +1SD) with job satisfaction at different levels of institutional betrayal (at -1SD and +1SD).

Both institutional betrayal and institutional courage uniquely predicted job satisfaction, with the whole model explaining 24% of the variance in job satisfaction scores. In the second step of the model, the interaction term was significant and improved the model’s predictive power, accounting for an additional 2% of the variance. See Fig 1 for a plot of the interaction and Table 4 for the model statistics. At low levels of institutional betrayal, job satisfaction is similar at all levels of institutional courage. However, at high levels of institutional *betrayal*, a high level of institutional *courage* buffers against associations with job satisfaction.

**Table 4:** Hierarchical multiple linear regressions models predicting employee outcomes.

Predictor	Step 1				Step 2				
	*B*	*SE*	*β*	ΔR^2^	*B*	*SE*	*β*	ΔR^2^	R^2^
**Job satisfaction**									
Gender—Cisgender Men	-0.05	0.87	-0.01		-0.27	0.86	-0.03		
Gender—Non-Binary/Transgender	-1.23	2.68	-0.15		-1.21	2.66	-0.14		
IBQ	-0.85***	0.13	-0.32		-0.85***	0.13	-0.32		
ICQ	-0.50***	0.08	0.34		0.50***	0.07	-0.34		
IBQ x ICQ	-	-	-	.237***	0.05**	0.02	0.12	.017**	.254***
**Affective commitment**									
Gender—Cisgender Men	-0.20	0.16	-0.14		-0.25	0.16	-0.17		
Gender—Non-Binary/Transgender	-0.19	0.50	-0.12		-0.18	0.49	-0.12		
IBQ	-0.15***	0.03	-0.31		-0.15***	0.02	-0.31		
ICQ	0.09***	0.01	0.32		0.09***	0.01	0.32		
IBQ x ICQ	-	-	-	.218***	0.01**	0.004	0.14	.023**	.241**
**Perceived gender bias**									
Gender—Cisgender Men	0.38*	0.17	0.24		0.33^	0.17	0.20		
Gender—Non-Binary/Transgender	-1.19*	0.53	-0.73		-1.19*	0.52	-0.73		
IBQ	-0.16***	0.03	-0.30		-0.16***	0.03	-0.30		
ICQ	0.07***	0.01	-0.23		0.06***	0.01	0.23		
IBQ x ICQ	-	-	-	.196***	0.01**	0.004	0.15	.029**	.225**
**One-year leaving intentions**									
Gender—Cisgender Men	0.26	0.36	0.08		0.28	0.37	0.09		
Gender—Non-Binary/Transgender	0.62	1.12	0.19		0.62	1.12	0.19		
IBQ	0.31***	0.06	0.30		0.31***	0.06	0.30		
ICQ	-0.13***	0.03	-0.22		-0.13***	0.03	-0.22		
IBQ x ICQ	-	-	-	.148***	-0.01	0.01	-0.03	.001	.149***
**Work withdrawal behaviors**									
Gender—Cisgender Men	4.28*	2.14	0.23		3.75^	2.12	0.20		
Gender—Non-Binary/Transgender	9.28	7.17	0.50		9.27	7.11	0.50		
IBQ	0.76*	0.33	0.13		0.76*	0.33	0.13		
ICQ	-0.44*	0.18	-0.14		-0.45*	0.18	-0.13		
IBQ x ICQ	-	-	-	.054**	0.13**	0.05	0.13	.021**	.075**
**Trust in management**									
Gender—Cisgender Men	-0.04	0.43	0.01		-0.01	0.43	-0.004		
Gender—Non-Binary/Transgender	-0.64	1.32	-0.18		-0.64	1.31	-0.18		
IBQ	0.02	0.07	0.02		0.02	0.07	0.02		
ICQ	0.10**	0.04	0.15		0.10**	0.04	0.16		
IBQ x ICQ	-	-	-	.024**	0.01	0.01	0.07	.005	.029**

***p < .001;

**p < .01;

*p < .05;

^*p* < .10.

Note: *n’s* for these analyses (*n* = 305) were less than 317 due to missing data on the ICQ and the IBQ that was not imputed (see Methods section). Abbreviations are as follows: IBQ (Institutional Betrayal Questionnaire), ICQ (Institutional Courage Questionnaire- Specific).

**Fig 2:**
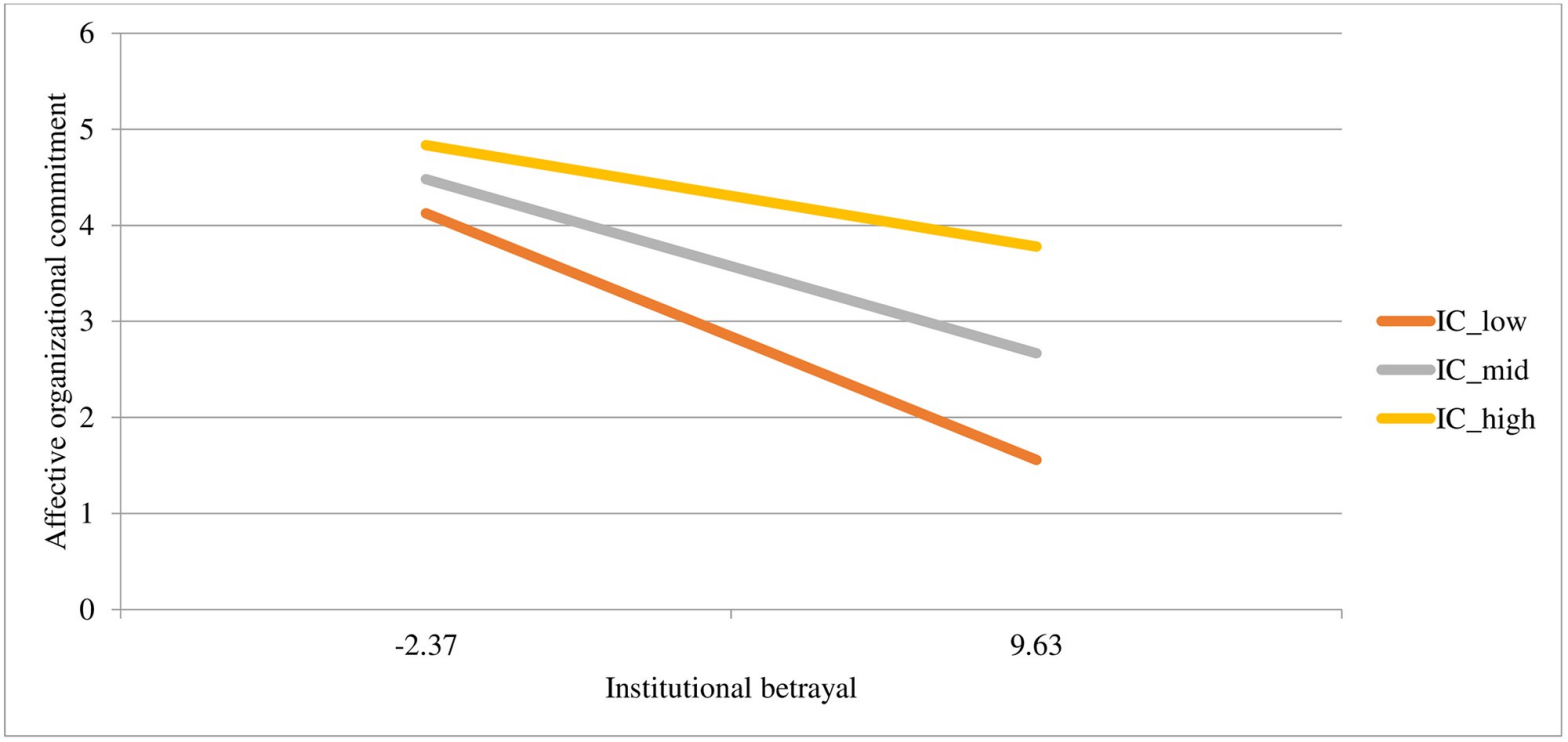
Associations between different levels of institutional courage (at -1SD, the mean, and +1SD) with affective organizational commitment at different levels of institutional betrayal (at -1SD and +1SD).

Both institutional betrayal and institutional courage uniquely predicted affective organizational commitment, with the whole model explaining 22% of the variance in scores. In the second step of the model, the interaction term was significant and improved the model’s predictive power, accounting for an additional 2% of the variance in affective organizational commitment. See Table 4 for model statistics and Fig 2 for a plot of the interaction. At low levels of institutional betrayal, affective organizational commitment is similar at all levels of institutional courage. However, at high levels of institutional *betrayal*, a high level of institutional *courage* buffers against associations with affective organizational commitment.

**Fig 3:**
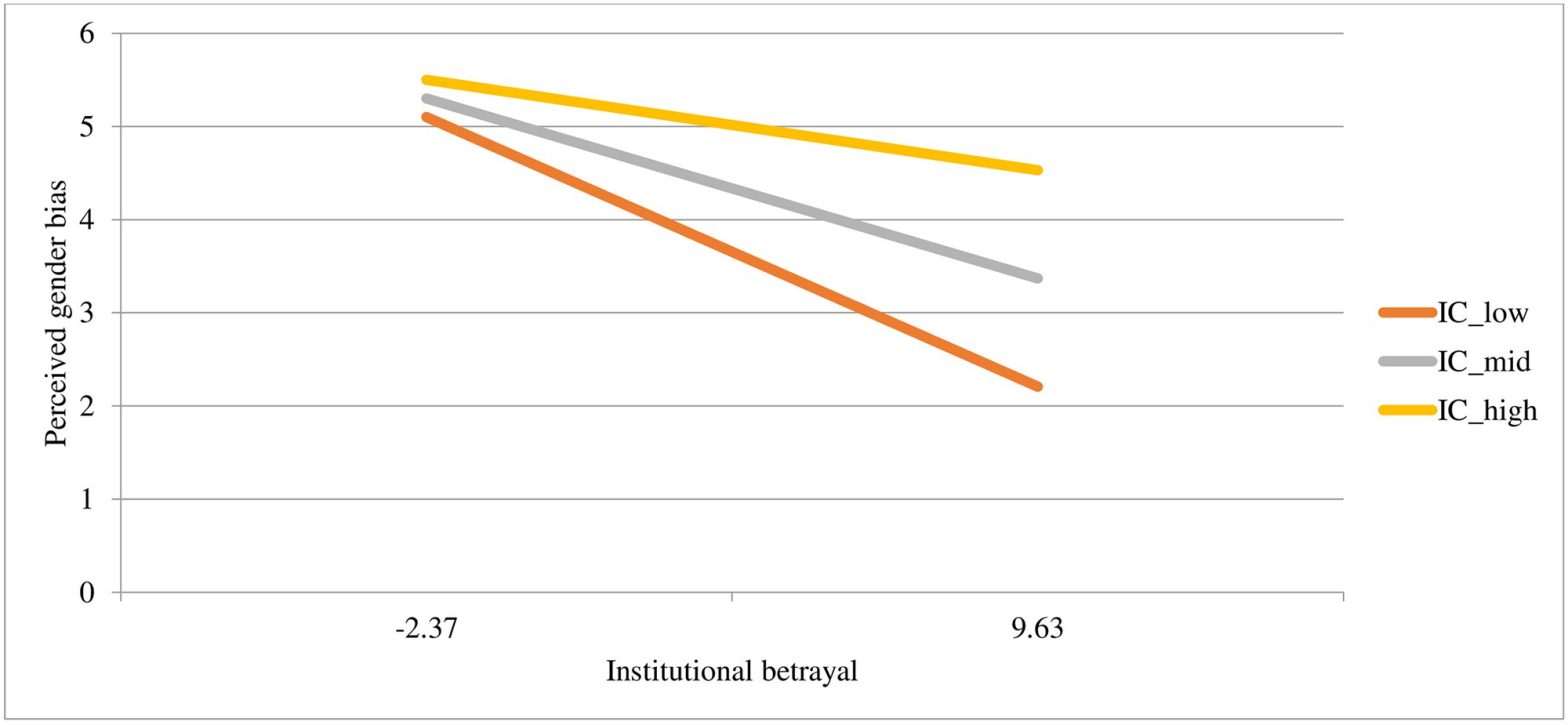
Associations between different levels of institutional courage (at -1SD, the mean, and +1SD) with gender bias at different levels of institutional betrayal (at -1SD and +1SD).

Both institutional betrayal and institutional courage uniquely predicted perceived gender bias, with the whole model explaining 20% of the variance in scores. In the second step of the model, the interaction term was significant and improved the model’s predictive power, accounting an additional 3% of the variance in perceived gender bias. See Table 4 for model statistics and Fig 3 for a plot of the interaction. At low levels of institutional betrayal, perceived gender bias is similar at all levels of institutional courage. However, at high levels of institutional *betrayal*, a high level of institutional *courage* buffers perceptions of gender bias.

Both institutional betrayal and institutional courage uniquely predicted one-year leaving intentions, with the whole model explaining 15% of the variance. In the second step of the model, the interaction term was not significant; institutional betrayal and institutional courage did not interact to statistically predict one-year leaving intentions (Table 4).

**Fig 4:**
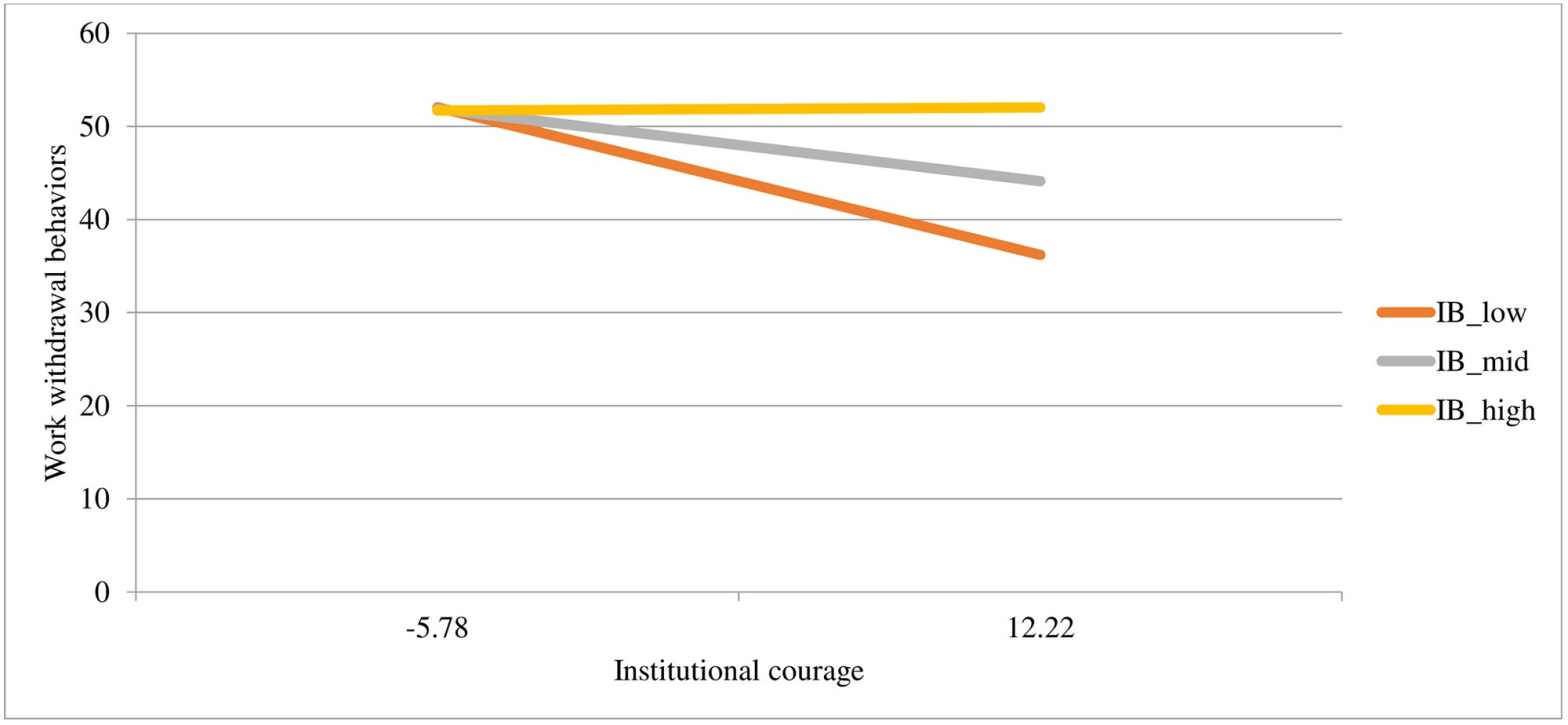
Associations of different levels of institutional courage (at -1SD, the mean, and +1SD) with work withdrawal at different levels of institutional betrayal (at -1SD and +1SD).

Both institutional betrayal and institutional courage uniquely predicted work withdrawal behaviors, with the whole model explaining 5% of the variance. In the second step of the model, the interaction term was significant and improved the model’s predictive power, accounting for and additional 2% of the variance in work withdrawal behaviors. See Table 4 for model statistics and Fig 4 for a plot of the interaction. As institutional courage increases, work withdrawal behaviors decrease, but only among employees who experience average or lower levels (-1*SD*) of institutional betrayal. Those employees who experience higher levels of institutional betrayal (+1*SD*) appear to have similar levels of work withdrawal behaviors regardless of the level of institutional courage.

Only institutional courage uniquely predicted trust in management, with the whole model explaining 2% of the variance. In the second step of the model, the interaction term was not significant; institutional betrayal and institutional courage did not interact to statistically predict trust in management (Table 4).

### Psychological and physical health outcomes—H2, H3, & H5

#### Correlational results—H2 & H3

Bivariate Pearson’s *r* correlations were computed to examine the initial associations between institutional betrayal, institutional courage, and employee psychological and physical health outcomes. Given the previous research linking a history of interpersonal trauma to the outcomes of interest, only individuals who had complete data for the BBTS were included in analyses. Institutional betrayal was significantly and positively correlated with each of the three outcomes of interest, including depression symptoms, anxiety symptoms, and somatic symptoms. Institutional courage was not significantly correlated with any of the psychological or physical health outcomes of interest. Histories of low, medium, and high betrayal trauma were each significantly and positively correlated with the psychological and physical health outcomes of interest. A history of high betrayal trauma had the strongest correlation with the psychological and physical health outcomes of interest (Table 5) and thus was used as a covariate in regression analyses.

**Table 5:** Means, standard deviations, and bivariate correlations between IBQ, ICQ-Specific, and employee psychological and physical health outcomes.

Variable	*M*	*SD*	1	2	3	4	5	6	7
1. Institutional betrayal	2.36	3.15							
2. Institutional courage	5.78	5.70	-.08						
3. Somatic symptoms	4.55	4.05	.24***	-.02					
4. Depression symptoms	5.04	4.52	.30***	-.11	.67***				
5. Anxiety symptoms	4.48	3.88	.23***	-.03	.63***	.81***			
6. BBTS—Low betrayal	1.02	1.38	.13*	.05	.27***	.23***	.20***		
7. BBTS—Medium betrayal	2.06	2.96	.28***	.04	.39***	.36***	.33***	.61***	
8. BBTS—High betrayal	1.23	1.56	.25***	.04	.41***	.43***	.39***	.49***	.71***

*Note*. *M* and *SD* are used to represent mean and standard deviation, respectively.

*p < .05.

**p < .01.

***p < .001.

#### Regression results—H5

In order to examine our hypotheses regarding an attenuation effect between institutional courage and institutional betrayal on psychological and physical health outcomes among those experiencing workplace sexual harassment, hierarchical linear multiple regression models were computed to examine the influence of institutional betrayal, institutional courage, and their interaction on each of the employee psychological and physical health variables of interest. Gender (with cisgender women as the reference group) and high betrayal trauma history were included as covariates. Each outcome was entered as the dependent variable. The mean-centered IBQ and ICQ-Specific variables were entered in the first step along with gender and the high betrayal trauma variable, followed by the IBQ-by-ICQ-Specific interaction term in the second step. Participants who did not complete either the IBQ or ICQ-Specific were excluded from the regression analyses.

Institutional betrayal, gender, and a history of high betrayal trauma uniquely predicted depression symptoms, with the whole model explaining 26% of the variance. Institutional courage was not a unique predictor of depression symptoms. In the second step of the model, the interaction term was not significant (Table 6).

**Table 6:** Hierarchical multiple linear regressions predicting psychological and physical symptoms.

Predictor	Step 1				Step 2				
	*B*	*SE*	*β*	ΔR^2^	*B*	*SE*	*β*	ΔR^2^	R^2^
**Depression symptoms**									
Gender—Cisgender Men	0.12	0.51	0.03		0.10	0.51	0.03		
Gender—Non-Binary/Transgender	4.13*	1.70	0.91		4.13*	1.70	0.91		
BBTS-HB	1.12***	0.17	0.39		1.12***	0.17	0.39		
IBQ	0.26**	0.08	0.18		0.26**	0.08	0.18		
ICQ	-0.08^	0.05	-0.10		-0.08^	0.05	-0.10		
IBQ x ICQ	-	-	-	.261***	0.004	0.01	0.02	.001	.262***
**Anxiety symptoms**									
Gender—Cisgender Men	-0.39	0.45	-0.10		-0.37	0.45	-0.09		
Gender—Non-Binary/Transgender	2.14	1.81	0.55		2.14	1.81	0.55		
BBTS-HB	0.88***	0.15	0.35		0.88	0.15	0.35		
IBQ	0.17*	0.08	0.14		0.17	0.08	0.13		
ICQ	-0.03	0.04	-0.04		-0.03	0.04	-0.04		
IBQ x ICQ	-	-	-	.188***	-0.01	0.01	-0.03	.001	.189***
**Somatic symptoms**									
Gender—Cisgender Men	0.15	0.46	0.04		0.05	0.46	0.01		
Gender—Non-Binary/Transgender	1.96	1.53	0.48		1.96	1.52	0.48		
BBTS-HB	0.95***	0.15	0.37		0.95***	0.15	0.37		
IBQ	0.95*	0.07	0.12		0.16*	0.07	0.13		
ICQ	0.16	0.04	0.01		0.01	0.04	0.01		
IBQ x ICQ	-	-	-	.187***	0.03*	0.01	0.11	.016*	.203***

*Note*.

***p < .001;

**p < .01;

*p < .05;

^*p <* .10.

The *n*’s used in analysis (*n* = 273) were not 317 due to missing data (some of which overlap) on the BBTS (33 data points), IBQ (6 data points), ICQ (11 data points) that were not imputed (see Methods section). Abbreviations are as follows: BBTS (Brief Betrayal Trauma Survey), IBQ (Institutional Betrayal Questionnaire), ICQ (Institutional Courage Questionnaire- Specific).

Institutional betrayal and a history of high betrayal trauma significantly predicted anxiety symptoms, with the whole model explaining 19% of the variance. Institutional courage was not a unique predictor of anxiety symptoms. In the second step of the model, the interaction term was not significant (Table 6).

**Fig 5:**
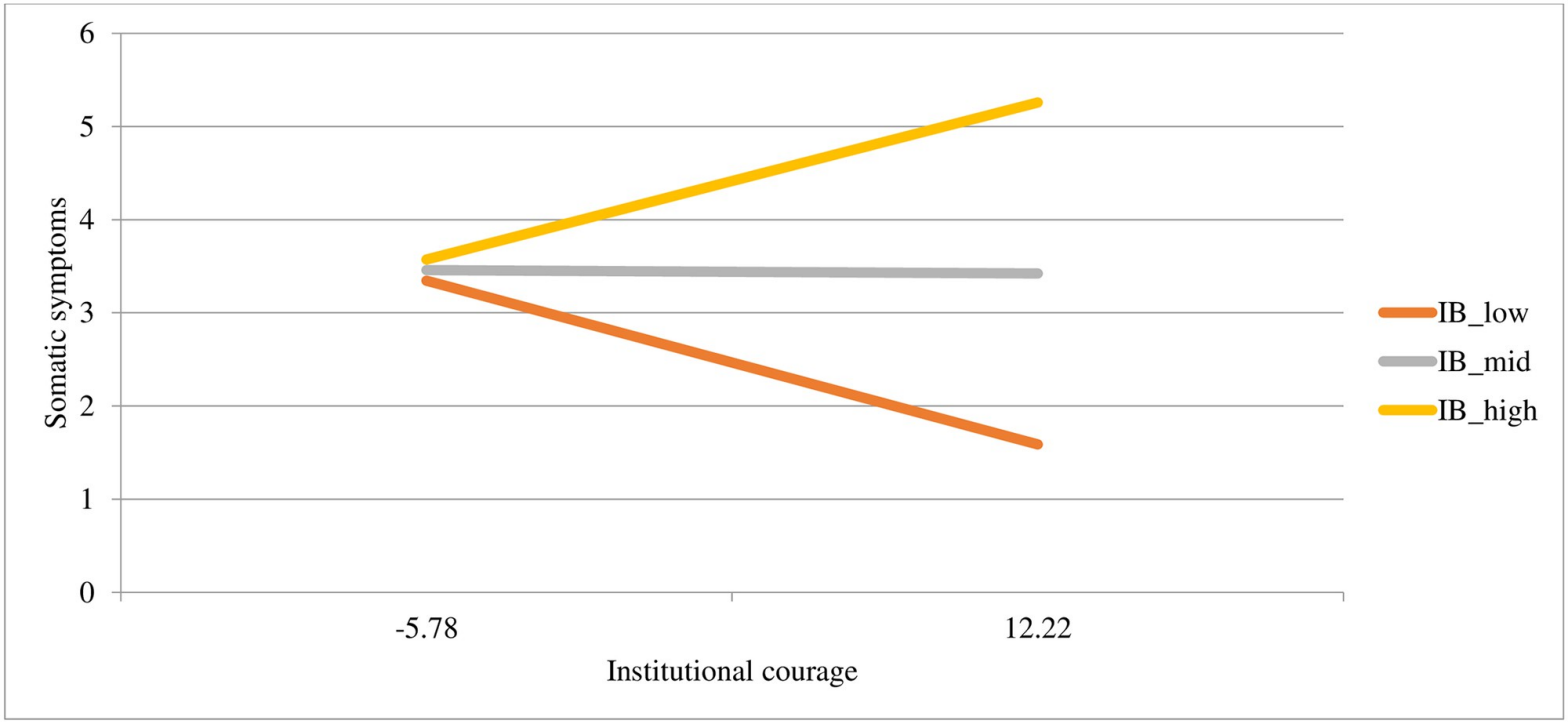
Associations between different levels of institutional courage (at -1SD, the mean, and +1SD) with somatic symptoms at different levels of institutional betrayal (at -1SD and +1SD).

Institutional betrayal and a history of high betrayal trauma uniquely predicted somatic symptoms, with the whole model explaining 19% of the variance. Institutional courage was not a unique predictor of somatic symptoms in step one. In the second step of the model, the interaction term was significant and improved the model’s predictive power, accounting for and additional 2% of the variance in somatic symptoms. To probe this interaction, we conducted a simple slopes analysis to determine if institutional courage would predict somatic symptoms at different values of institutional betrayal. At +/– 1 standard deviation of institutional betrayal, institutional courage remained a non-significant predictor of somatic symptoms. However, at +/– 1.5 standard deviations of institutional betrayal, institutional courage became a significant predictor of somatic symptoms in the second step of the model. At very low levels of institutional betrayal, institutional courage is associated with decreased somatic symptoms. Conversely, at very high levels of institutional betrayal, institutional courage is associated with increased somatic symptoms (+1.5*SD* of institutional betrayal, institutional courage is significant, *p* = .036; at -1.5*SD* of institutional betrayal, institutional courage is significant, *p* = .034). See Table 6 for model statistics and Fig 5 for a plot of the interaction.

## Discussion

The aim of the current study was to investigate how institutional courage and institutional betrayal following workplace sexual harassment is related to a variety of outcomes among employees. Of participants who experienced workplace sexual harassment, nearly 55% also experienced institutional betrayal, and 76% also experienced institutional courage. We hypothesized that both institutional courage and institutional betrayal would be significantly associated with both workplace employee outcomes and physical and mental health outcomes. We also hypothesized that institutional courage would interact with institutional betrayal, such that institutional courage would attenuate the negative relationship between institutional betrayal and negative outcomes.

Our hypotheses regarding employee outcomes were largely supported. There were significant correlations observed between institutional betrayal and the employee workplace outcomes of interests except for one (trust in management), and there were also significant correlations observed between institutional courage and the employee workplace outcomes of interest. In many of the multiple regression models, an examination of the interaction between institutional betrayal and institutional courage reveals that as the amount of institutional betrayal one experiences increases, institutional courage appears to buffer against the influence of institutional betrayal. This is important for intervention and countering of institutional betrayal. In other words, it is a problem that could be ameliorated and countered with courageous actions by the institution.

There were also three employment outcomes with non-significant interactions that are important to consider. The first of these is employee one-year leaving intentions. Institutional betrayal was associated with higher intentions to leave, whereas institutional courage was associated with lower intentions to leave. This is in line with the results above suggesting institutional betrayal and courage are associated with organizational commitment and job satisfaction. Future research should examine this longitudinally to further explicate the interplay between intentions to leave the institution, the costs/necessity calculus of remaining or leaving, and employees’ actual actions (i.e., remaining or leaving). Lastly, institutional courage was associated with greater trust in management, although institutional betrayal was not associated with this particular outcome. Although the association between institutional courage and trust in management does cohere with the above results on employee satisfaction and engagement, the lack of an association with institutional betrayal is a departure from this set of results, as well as from one study on patient trust in healthcare systems [53]. This also represents an area for further study—to determine if level of trust in management changes over time with experiences of institutional betrayal and courage.

Our hypotheses regarding psychological and physical health outcomes were partially supported. Significant positive correlations were observed between institutional betrayal and the outcomes of interest, whereas no significant correlations were observed between institutional courage and the outcomes of interest. Subsequent linear regression models revealed that only institutional betrayal was uniquely associated with higher depression, and anxiety symptoms. Additionally, the interaction terms were not significant for these two outcomes, suggesting that institutional betrayal and institutional courage do not interact with each other in their effect on depression, or anxiety symptoms.

Our results with respect to somatic symptoms are also interesting. In the regression model, institutional betrayal was uniquely associated with a significant increase in somatic symptoms, whereas institutional courage was not a significant predictor. However, at lower levels of institutional betrayal, institutional courage was associated with decreased somatic symptoms, whereas at higher levels of institutional betrayal, institutional courage was associated with increased somatic symptoms. This is contrary to our predictions that institutional courage would act as a buffer for the negative effects of institutional betrayal. A number of things could be driving this relationship. First, it could be that at very high levels of institutional betrayal, the degree of betrayal may be so great that, when there are instances of institutional courage, they may be interpreted as disingenuous, given the existing degree of betrayal. Second, because the institutional betrayal data only extend to 2.1*SD* above the mean, it is possible that because the *n* decreases as one approaches the tails of the distribution of institutional betrayal, outliers could be driving these effects. Given that the model containing somatic symptoms was the only one among the psychological and physical health outcomes that had a significant interaction effect, somatic symptoms present an area for further investigation.

### Practical and clinical implications

The results presented here suggest that both institutional betrayal and institutional courage are not unusual events in the context of workplace sexual harassment. While it is heartening to see that just over three-quarters of employees who experienced sexual harassment also experienced institutional courage, it is discouraging—although unsurprising—to see that over half of employees experienced institutional betrayal. In fact, these rates of institutional betrayal are higher than those observed in the context of sexual assault in some studies (e.g., [18, 19]). These findings thus have implications for professionals and practitioners in a wide variety of fields, such as management, human resources, and human capital management. Education about the frequency and forms of institutional betrayal and institutional courage is the first step to identifying these phenomena, as well as their effects within institutions at both the macro/structural and micro/individual levels.

As aforementioned, our results suggest multiple domains of impact. In previous research on institutional betrayal, the focus has largely been—and with good reason—on the negative psychological and physical health effects that result from these types of experiences (for a brief review, see [54]). Consider this the “first argument” for reducing institutional betrayal and increasing institutional courage; in other words, because institutional betrayal is associated with psychological and physical harm, it is “worth” reducing institutional betrayal and increasing institutional courage. It is “worth” doing so in the sense that reducing harm is a compassionate, moral act. Much in the way that research on institutional betrayal in the healthcare system has focused on disengagement from healthcare [23, 53], this study similarly presents an additional, second argument both regarding the effects of institutional betrayal, that of negative employee workplace outcomes and institutional health, and for replacing institutional betrayal with institutional courage. Although we did not explore possible downstream economic and institutional effectiveness outcomes (i.e., the degree to which an organization achieves its stated goals, which may include profit benchmarks or production targets) as associated with institutional betrayal, institutional courage, and employee outcomes, it is important to recognize that the employee workplace and employment-related outcomes examined in this study can and do have a substantial impact on organizations ([40], e.g., [55]).

### Limitations

The results of this study should be interpreted in light of its limitations. First, we collected data at only one timepoint, which limits any conclusions related to direct causality. This was in line with the aim of the study, which included characterizing institutional courage and institutional betrayal with respect to workplace sexual harassment—the first empirical examination of these phenomena in this context, and the first empirical examination of institutional courage. This design does limit our ability to examine the time-course of events, especially as it is likely that institutional betrayal and institutional courage occur over time and at different points following an incident of sexual harassment. A longitudinal design would be particularly useful for examining both employee workplace outcomes as well as psychological and physical health outcomes over time. Second, we had small cell sizes for non-gender-binary individuals (i.e., those that do not identify as “man” or “woman”). This limits the generalizability of our results to individuals who are gender minorities, a group who has been found to experience very high rates of sexual harassment and other types of victimization [56, 57]. Similarly, future investigations may benefit from oversampling individuals who identify as racial and ethnic minorities, given that racial and ethnic minorities often experience greater rates, and more severe types, of both workplace sexual and general harassment in the workplace [58–60]. Third, because of the exploratory phase of the research, we cast a wide net of variables measured. We acknowledge that this may have inflated Type I error, with some overlap in the outcome variables.

### Future directions

There are many avenues for future research on institutional betrayal and institutional courage. First, examining the rates at which institutional courage and institutional betrayal occur following workplace sexual harassment by employment industry and sector would add to the current findings. Additionally, examining institutional courage and institutional betrayal within one organization could be useful for examining multilevel effects, such as individuals nested within teams nested within units. Such models would provide a better understanding of these effects at different levels within organizations, which in turn could further delineate targets for intervention.

Interventions to replace institutional betrayal with institutional courage should start with education—by training employees, supervisors, human resources professionals, executives, and so on—to first identify institutional betrayal and institutional courage when they happen. Institutions can enact policies and procedures designed to reduce institutional betrayal (e.g., allowing victims to have a say in how their case is handled, such as opting for victim-centered policies rather than policies of mandatory reporting/compelled disclosure; [61]), and promote institutional courage (e.g., an incentive structure that rewards reporting sexual harassment). In either of these forms of intervention—and many others exist too—data should be gathered throughout the process of intervention design, implementation, and dissemination.

## Conclusion

Underlying all research on institutional betrayal and institutional courage is the idea that how one responds to a negative event—whether sexual harassment, sexual assault, and other types of victimization—is often as important or more important for future outcomes as the original event itself. In other words, it’s not only about what happens; it’s also about what happens next. In this study, institutional betrayal and institutional courage appear to have a tangible association with employee workplace and health outcomes. Furthermore, institutional courage appears to attenuate negative outcomes in both the employee workplace and health domains.

While we once again find that institutional betrayal is harmful, this study indicates that institutional courage can buffer against those harms. The ultimate goal of this research is to eliminate institutional betrayal at all levels of institutions by replacing it with institutional courage. The current study provides a starting point to achieving that goal by introducing a new measure of institutional courage to be used in future investigations and by reporting findings that demonstrate the power of institutional courage with respect to workplace sexual harassment.
